# Emerging Technologies in Granulation: Process Analytical Technologies and Modeling Approaches in Pharmaceutical Development and Manufacturing

**DOI:** 10.3390/pharmaceutics18101286

**Published:** 2026-10-10

**Authors:** Panagiotis Pentsis, Maria Chountoulesi, Eirini Christodoulou, Natassa Pippa

**Affiliations:** 1 Master’s Program “Development of New Drugs: Research, Launch and Access”, School of Medicine, National and Kapodistrian University of Athens, 11527 Athens, Greece; ppentsis@med.uoa.gr; 2 Section of Pharmaceutical Technology, Department of Pharmacy, School of Health Sciences, National and Kapodistrian University of Athens, Panepistimioupolis Zografou, 15771 Athens, Greece; 3 Laboratory of Biopharmaceutics & Pharmacokinetics, Department of Pharmacy, School of Health Sciences, National and Kapodistrian University of Athens, 15771 Athens, Greece; e_christod@pharm.uoa.gr

**Keywords:** granulation, Process Analytical Technologies, solid dosage forms, process parameters, data modeling, twin-screw wet granulation, Near-Infrared Spectroscopy, fluid-bed granulation, Quality by Design, continuous manufacturing

## Abstract

In the present review, 32 studies, published between 2020 and 2026, were reviewed to identify recent advances in pharmaceutical granulation. Emphasis is placed on the integration of real-time monitoring technologies, recent Process Analytical Technologies (PATs) approaches, the relationships between critical process parameters (CPPs) and critical quality attributes (CQAs) affecting the product, and continuous manufacturing, as well as the implementation of modern data processing models. Increased attention was paid to continuous granulation using twin-screw extrusion, alongside the use of spectroscopic technologies for monitoring the production continuum. Finally, novel modeling approaches are discussed, revealing a transition from solely empirical methods toward hybrid models that combine data analysis tools with mechanistic process understanding and Artificial Intelligence (AI)-driven applications, yielding a new era with the transformation of both batch and continuous manufacturing. The study highlights the integration of PATs, modeling and QbD to enhance process understanding and control, but also challenges from a regulatory framework perspective that resulting from these technological advancements.

## 1. Introduction

In the manufacturing process of solid dosage forms, raw materials must undergo specific procedures to acquire the desired properties for compression or encapsulation [1]. The principal downstream method to achieve this on a large scale is the granulation process, which refers to the formation of agglomerated particles with or without the presence of a liquid binder [2].

There are three distinct categories of granulation: wet, dry and melt. During wet granulation, particles of the active pharmaceutical ingredient (API) and excipients are agglomerated by the addition of a granulating liquid, which may contain a dissolved or dispersed binder. The liquid wets the powder particles and forms liquid bridges between them, generating capillary forces that promote particle adhesion and agglomeration, while the binder can contribute to the solid bridges/bonds that remain after drying. As granulation proceeds, particles can coalesce and form larger, denser granules. During subsequent drying, the liquid evaporates and the binder, along with any other excipients being contained in the selected liquid, solidify or become more concentrated, contributing to the formation of solid interparticle bonds and mechanically stable granules with improved flowability and compressibility [3]. Wet granulation remains fundamental for pharmaceutical manufacturing, as it can, after careful process design and control, modify and substantially improve the powder flowability, bulk density and compressibility profiles for granules, ensure content homogeneity of the product and minimize fines by incorporating them into larger particles, while reducing dust generation and segregation. Consequently, it is particularly advantageous for formulations containing low-dose APIs, cohesive powders, or blends with poor flow characteristics. However, mechanical complexity and high energy costs remain the main drawbacks, coupled with the fact that the different stages involved can lead to product loss. Moreover, APIs that are sensitive to heat exposure present a poor candidate for wet granulation as they cannot withstand the high temperatures of the drying process. High-shear granulation and fluid-bed granulation are the most common wet granulation technologies as batch processes, while twin-screw wet granulation has emerged as a key enabling technology for continuous manufacturing [4,5,6]. High-shear granulation provides rapid densification and short processing times, while fluid-bed granulation combines granulation and drying into a single unit operation, thereby reducing processing time and equipment requirements [7].

In dry granulation no liquid binder is used. Instead, the blended materials are subjected to high compression forces by passing through a roller compactor or slugging, followed by milling to produce granules. Dry granulation is suitable for heat or moisture sensitive APIs and is generally more energy-efficient due to the use of fewer manufacturing steps, does not necessarily require the use of binder and thus being able to contribute to product quality and stability. Compared with wet granulation, it involves fewer processing steps, which can reduce processing time, energy consumption, and manufacturing costs. The absence of a wetting and drying stage may also contribute to improved product stability by minimizing exposure to moisture and heat. Dry granulation may be applicable to high-dose formulations and is well suited to continuous manufacturing; however, its feasibility depends on the compressibility and compactability of the API and powder blend. In particular, high API loading may limit roller compaction when the drug substance exhibits poor compaction properties [8]. The major challenges of dry granulation include the excessive production of fine particles that can negatively affect granule quality and the difficulty in modifying process parameters to achieve consistent product attributes and may lead to poor flowability, segregation, and inconsistent tablet properties [9]. Slugging, roll compaction process and pneumatic dry granulation are different categories of dry granulation, while optimizing ribbon microstructure, and implementing PAT-based monitoring are engaged to achieve more consistent product quality [7].

Finally, melt granulation is employed for poorly water soluble and moisture sensitive APIs by adding them to a molten binder or a solid binder, usually a thermoplastic polymer or a lipid-based excipient, which melts between a specific temperature range, thereby promoting particle agglomeration without the use of solvents. Upon cooling, the binder solidifies, forming stable granules with improved mechanical strength. By dispersing the API within a molten polymeric or lipid matrix, the process can improve drug wettability, reduce particle size, and in some cases promote the formation of amorphous solid dispersions, leading to increased dissolution rates. Common binders used in melt granulation include polyethylene glycols (PEGs), polyvinylpyrrolidone (PVP), polyethylene oxide (PEO), poloxamers, glyceryl behenate, and other waxes and lipid excipients, with the choice of binder depending on the melting temperature, compatibility with the API, and the desired release characteristics of the final dosage form [10,11]. Although it is a viable alternative for processing moisture sensitive materials, proper polymer selection and heat tolerance are two major factors to be considered before developing a melt granulation process, because its applicability to heat-sensitive APIs or excipients is limited by the temperatures required to melt the binder [11].

Unlike conventional wet granulation, the melt granulation eliminates the need for a liquid phase and a subsequent drying step, resulting in a shorter manufacturing process, reduced energy consumption, and lower production costs, increasing process sustainability. In addition, the absence of solvents makes the technique particularly attractive for moisture-sensitive formulations and reduces environmental concerns associated with solvent handling and disposal. Despite these advantages, successful process development requires careful selection of the binder and optimization of processing conditions to avoid thermal degradation of heat-sensitive APIs or excipients. Furthermore, the lower viscosity of the molten binder, the high shearing forces, the smaller binder droplet size can provide a uniform binder distribution, and are essential for producing granules with consistent size, density, and mechanical properties [7].

Among the available processing platforms for melt granulation, the twin-screw extrusion has emerged as an advantageous one. In twin-screw melt granulation, powders are continuously fed into a heated twin-screw extruder, where the binder melts and promotes particle agglomeration through the combined effects of heat, shear, and mixing. The resulting agglomerates are subsequently cooled and collected without requiring a separate drying step. Compared with conventional batch melt granulation, the use of twin-screw extrusion as a continuous processing platform offers potential advantages, including continuous operation, reduced processing time, improved process control and reproducibility, enhanced scalability, and greater batch-to-batch consistency. The modular design of twin-screw extruders also allows key process parameters, such as screw configuration, barrel temperature profile, feed rate, and screw speed, to be adjusted according to the formulation and desired product characteristics. Optimization of these parameters can influence granule properties, including particle size distribution, density, porosity, and mechanical strength, thereby facilitating the production of granules that meet predefined critical quality attributes (CQAs). In addition, the continuous nature of twin-screw extrusion facilitates integration with downstream unit operations, such as milling and tableting, supporting process intensification and scale-up [12,13].

Although granulation has been a well-defined method in terms of mechanical instrumentation, with each type having its own distinct configuration of interconnecting operational units, the focus has shifted in finding new ways to use it more effectively in drug manufacturing, according to the principles of Quality by Design (QbD), by identifying the relevant critical quality attributes (CQAs) and critical process parameters (CPPs) (Table 1), as well as the establishment of a design space within which acceptable product quality can be consistently achieved. The selection of an appropriate granulation method depends on the physicochemical properties of the active pharmaceutical ingredient (API), excipient characteristics, desired product performance, manufacturing scale, and regulatory considerations. According to the QbD framework, product quality should be built into the process through systematic understanding rather than relying solely on end-product testing. At the same time, the pharmaceutical industry aims to eliminate the need for batch production and tip the balance towards a continuous manufacturing model capable of ushering an era of seamless and uninterrupted production. Despite the numerous in-depth publications on individual granulation techniques, recent studies do not present a unified front towards the integration of QbD principles, real-time monitoring tools and modeling approaches across different granulation processes.

Critical material attributes play a fundamental role in determining granulation performance. Important CMAs include particle size distribution, particle morphology, bulk and tapped density, surface area, wettability, moisture content, and excipient functionality. The particle size of active pharmaceutical ingredients (APIs) and excipients influence nucleation, granule growth, and final granule size distribution. Similarly, material properties such as hygroscopicity and surface energy can affect liquid distribution and granule formation mechanisms. Binder properties, including viscosity, concentration, and molecular weight, are also recognized as critical factors influencing granule strength and stability.

Critical process parameters vary depending on the granulation technology employed. In high-shear wet granulation, commonly investigated CPPs include impeller speed, chopper speed, binder addition rate, liquid-to-solid ratio, spray rate, granulation time, and wet massing duration. These parameters directly influence granule growth mechanisms such as nucleation, coalescence, consolidation, and breakage. In fluid-bed granulation, atomization pressure, inlet air temperature, spray rate, and airflow rate are among the most influential variables. For twin-screw wet granulation, screw speed, feed rate, barrel configuration, liquid addition rate, and residence time are considered critical process parameters due to their impact on mixing efficiency and granule formation [14,15].

**Table 1:** Typical critical material attributes, process parameters and quality attributes of granulation processes.

Critical Material Attributes	Critical Process Parameters	Critical Quality Attributes
***High/Low Shear***		
API particle size distribution	Water amount	Granule size distribution
API morphology	Pre-blending time	Bulk and tapped density
Excipient particle size	Fill level–occupancy	Moisture content (LOD)
Binder type and concentration	Order of addition	Flowability
Binder viscosity	Mixing time	Compressibility
Moisture content of raw materials	Impeller and chopper (speed, configuration and location)	Blend/content uniformity
Powder flowability	Wet massing time	Tablet hardness
Compressibility	Vessel temperature	Friability
API/excipient ratio	Granulation time	Dissolution profile
API polymorphic form	Spray nozzle type and location	Content uniformity
Bulk and tapped density	Liquid/solid ratio	API Solid-state form (crystallinity/amorphicity)
Cohesive/adhesive properties	Binder addition method	Granule brittleness
Solid form/polymorph	Binder temperature	
Hardness/plasticity	Geometry of HS	
Viscoelasticity	Top- or bottom-driven	
Electrostatic properties		
***Fluid Bed***		
API particle size	Inlet air (Flow rate, volume, temperature)	Granule size distribution
Powder wettability	Binder spray rate	Moisture content
Binder properties (type, concentration, viscosity)	Air flow rate	bulk/tapped density
Spray solution viscosity	Atomization air pressure	Flowability
Excipient particle size	Exhaust air temperature	Granule strength
Moisture sensitivity	Blending time	Blend/content uniformity
Powder flowability	Spray nozzle (type, quantity, pattern, configuration)	Dissolution
Compressibility	Binder addition method	tablet hardness
API/excipient ratio	Binder fluid temperature	Friability
API polymorphic form	Filter (properties, size)	API Solid-state form (crystallinity/amorphicity)
Bulk and tapped density	Product temperature	Compressibility
	Shaking intervals	
	Type of base plate	
	Starter pellets size	
***Spray Dying***		
API particle size distribution	Inlet temperature	Particle/granule size distribution
API morphology/crystal form	Spray flow rate	Moisture content
Moisture content	Solids ratio (%)	Bulk/tapped density
Excipient particle size	Solvent system (organic/aqueous)	Flowability
Binder properties (type, concentration, viscosity)	Atomization	Blend/content uniformity
Bulk and true density	Spray nozzle	Tablet hardness
Powder flowability	Surface tension	Friability
Compressibility		Dissolution profile
Drug–excipient compatibility		Assay/content uniformity
Feed viscosity		Solid-state form (crystallinity/amorphicity)
Powder wettability		Residual solvent
Spray solution viscosity		
Solvent composition		
API T_m_		
Polymer T_g_ or T_m_		
***Roller Compactor***		
API particle size distribution	Feed rate	Particle/granule size distribution
API morphology/crystal form	Lubricant flow rate	Moisture content
Moisture content	Roller gap	Bulk/tapped density
Excipient particle size	Compression pressure	Flowability
Binder properties (type, concentration, viscosity)	Roll speed	Blend/content uniformity
Bulk and true density	Density of ribbon	Tablet hardness
Powder flowability		Friability
Compressibility		Dissolution profile
Drug–excipient compatibility		Assay/content uniformity
Particle hardness		Solid-state form (crystallinity/amorphicity)
Excipient deformation behavior (plastic/brittle)		Ribbon density
Lubricant concentration		Ribbon strength
		Fines level
***Melt Extrusion***		
API particle size distribution	Screw shape	Particle/granule size distribution
API morphology/crystal form	Screw speed	Moisture content
Moisture content	Zone number/temperature	Bulk/tapped density
Excipient particle size	Feed rate (API, excipients, liquid)	Flowability
Binder properties (type, concentration, viscosity)		Blend/content uniformity
Bulk and true density		Tablet hardness
Powder flowability		Friability
Compressibility		Dissolution profile
Drug–excipient compatibility		Assay/content uniformity
API thermal stability		Solid-state form (crystallinity/amorphicity)
polymer grade and molecular weight		Extrudate dimensions
API-polymer miscibility		
drug loading		
polymer viscosity		
plasticizer concentration		
***Twin-Screw (Continuous Granulation/Extrusion)***		
API T_m_	Screw speed	Particle/granule size distribution
Polymer T_g_ or T_m_	Number of die holes	Moisture content
API particle size distribution	Fill level	Bulk/tapped density
API morphology/crystal form	Feed rate	Flowability
Moisture content	Liquid/solid ratio	Blend/content uniformity
Excipient particle size	Screw configuration/type/size	Tablet hardness
Binder properties (type, concentration, viscosity)	Processing temperature	Friability
Bulk and true density	Melt viscosity	Dissolution profile
Powder flowability	Torque	Assay/content uniformity
Compressibility		Solid-state form (crystallinity/amorphicity)
Drug–excipient compatibility		Residence time distribution
Polymer viscosity (if extrusion)		

**Figure 1:**
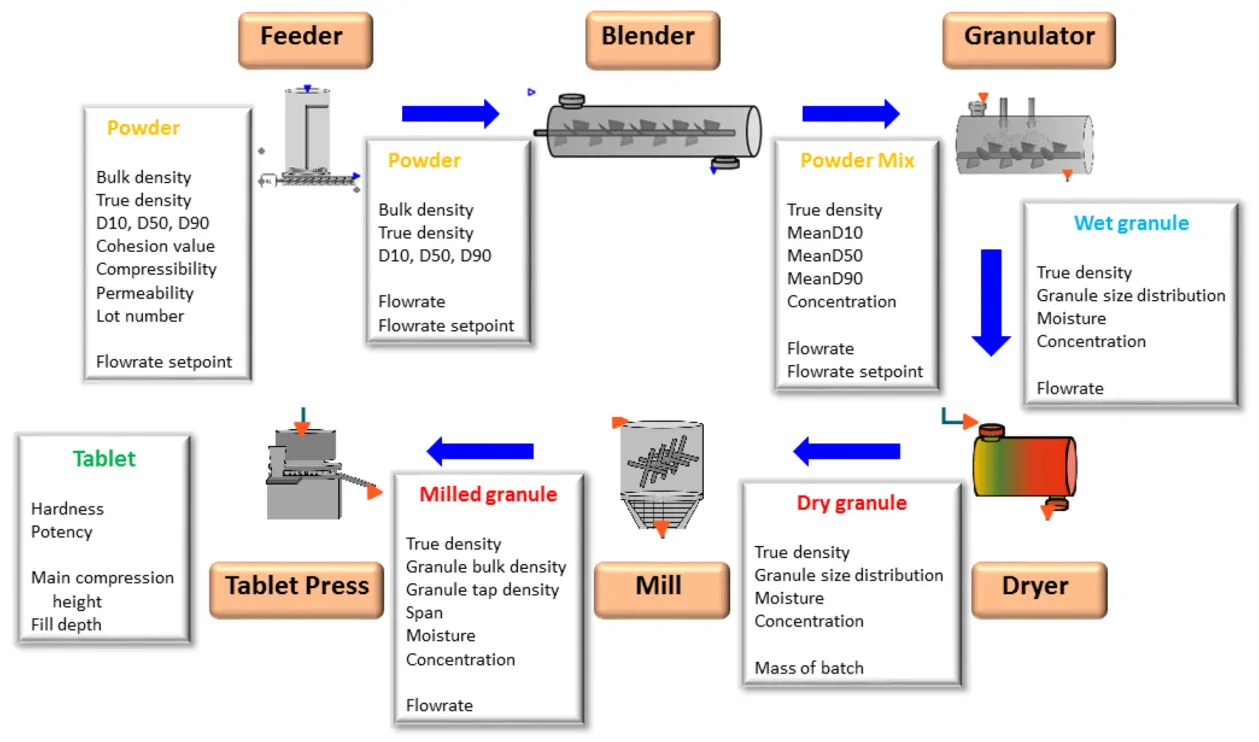
Schematic showing transfer of information between units in wet granulation, in a flowsheet model, based on models that are developed from experimental runs on units included in the continuous line using the same formulation and materials. The flowsheet incorporates models of the intermediate unit operations, including the powder blend feeder, granule feeder, and granule blender, as well as the associated material transfer lines. The resulting integrated model enables the simulation of the continuous process and the evaluation of how changes in process variables propagate through the manufacturing line and influence intermediate and final product properties, assessing the sensitivity of intermediate and final product CQAs to CPPs variables that should be identified. Controlling of the process parameters towards maintaining the required CQAs is essential throughout the process. Adapted from Metta et al. [16], with Creative Commons CC BY 4.0 open-access license.

The quality of pharmaceutical granules is typically assessed through a range of critical quality attributes. Granule size distribution (after the step of sizing) is one of the most important CQAs because it influences flowability, die filling, compressibility, and dissolution performance. Other commonly evaluated CQAs include granule density, porosity, water content/LOD, mechanical strength, friability, flow properties, and content uniformity. In many cases, the final product quality, including tablet hardness, disintegration time, and dissolution behavior, is strongly linked to granule characteristics generated during upstream processing (Figure 1). It is essential to control the process parameters and maintain the QAs of granules upon scale-up step of wet granulation [15].

Following a drug product’s active substance release, changes and modifications are dictated by the Food and Drug Administration’s (FDA) Scale-Up and Post-Approval Changes (SUPAC) guidelines, a comprehensive document that outlines all recent requirements regarding the manufacturing of pharmaceutical products. The FDA defines all aspects of the granulation process and categorizes potential changes, based on the impact they may have on crucial product attributes, into three levels: major (Level 3), moderate (Level 2), and minor (Level 1). This framework aims to facilitate the adoption of new approaches in pharmaceutical manufacturing and align current techniques with QbD, incorporating the use of CQAs and CPPs [17].

Quality by Design (QbD) is a systematic, science- and risk-based approach to pharmaceutical development that emphasizes predefined quality objectives, understanding of the relationships between material attributes and process parameters, and the development of a robust control strategy to consistently achieve the desired product quality [18]. The FDA and the European Medicines Agency (EMA) have opted for the implementation of QbD which is evident by the several guidance documents issued by the International Council for Harmonization (ICH), including ICH Q8 [19], Q9 [20], Q10 [21], Q11 [22], and Q12 [23]. QbD aims to reduce the cost and time of pharmaceutical production by offering an adaptable real-time manufacturing framework based on in-line control of the process parameters and product attributes [18].

According to ICH Q8, CQAs are the physical, chemical, biological, or microbiological product traits that directly affect drug quality. They can refer to raw materials, intermediates (e.g., granules) or final dosage forms. The CQAs have been identified through product experimentation and process understanding, and their importance depends on the impact that their variations have on the final product [19,24]. In contrast, CPPs have a direct link to the input parameters used to monitor drug processing. They are the values that describe the conditions under which the product is manufactured, and their ranges must be established to guarantee product quality [19].

The aim of this narrative review is to analyze new approaches in granulation across the varying types of the process. The focus is on how novel research implements modern tools for monitoring the pharmaceutical production of oral dosage forms according to the guidelines of international organizations. Recent developments are also covered, focusing on mechanistic modeling, computational simulations, and digital process control to better understand granule growth mechanisms and optimize manufacturing efficiency while minimizing product variability. Finally, the present paper strives to uncover the complex relationships between product attributes and process parameters through the use of currently available modeling techniques.

## 2. Methodology

A thorough literature search was conducted on the PubMed database, which focused on peer-reviewed articles published between the years 2020 and 2026. The research included combinations of keywords such as pharmaceutical granulation, solid dosage forms, Process Analytical Technologies, Quality by Design, critical quality attributes, critical process parameters and process modeling, while relevant reviews were also analyzed to uncover additional relevant studies. The criteria for inclusion involved studies with experimental or industrial applications of wet, dry and melt granulation processes that offered significant evidence for the relationship between process parameters and product attributes, including adjacent/enabling upstream or downstream operations when they provided directly relevant information about granulation, process monitoring, material behavior, or downstream processing.

**Figure 2:**
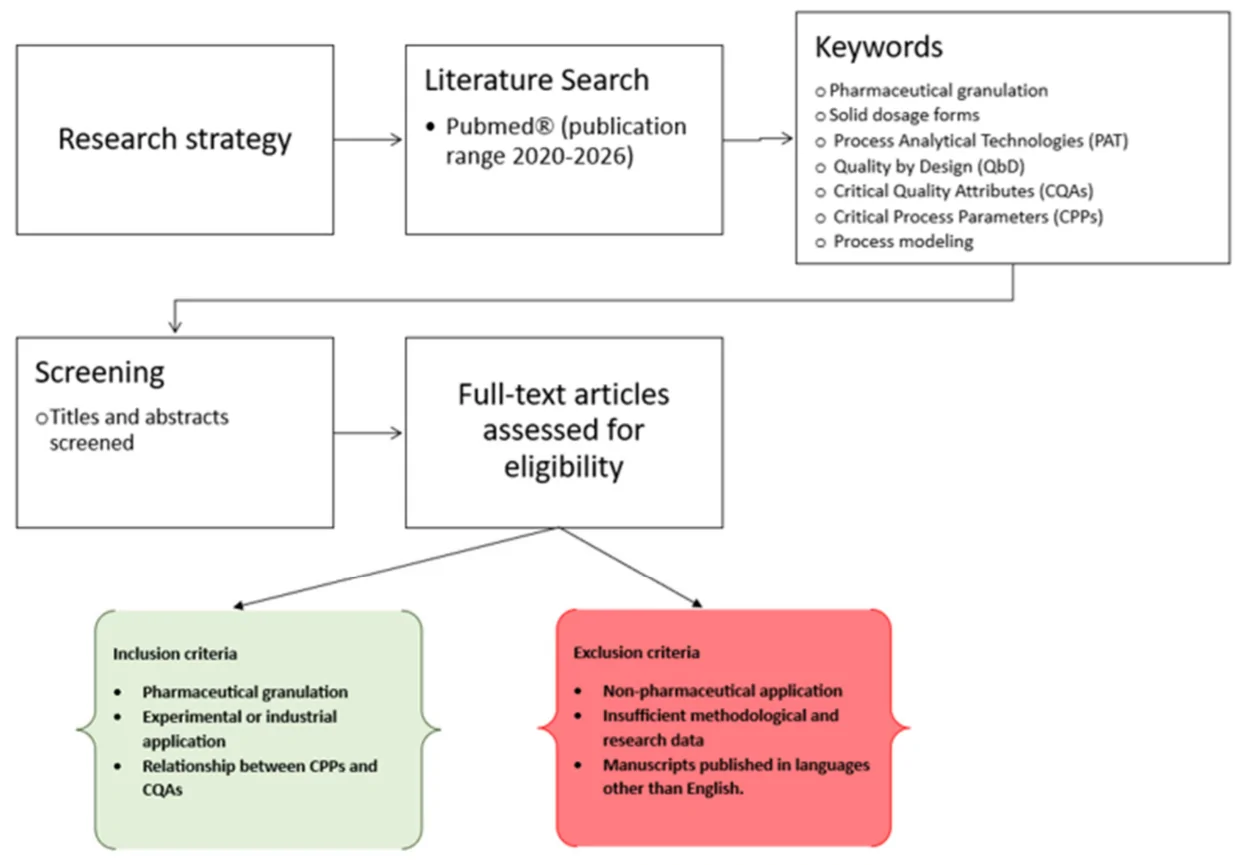
Case study selection flow diagram for the literature evaluated in the present review.

Additionally, the research manuscripts were evaluated on the basis of physicochemical and material properties of the APIs, which led to the formulation strategy, selection of excipients, and consideration of the process of manufacturing. In other words, the aforementioned criteria used to select an appropriate manufacturing process, the methodology used to identify and screen the final studies, and how the relevant CMAs, CPPs, and CQAs were identified. CMAs were identified when a material property was demonstrated or scientifically justified to influence processability, granulation behavior, or a relevant product quality attribute. CPPs were identified based on evidence that variation in a process parameter could have a significant influence on one or more product quality attributes. CQAs were identified from the quality attributes evaluated in the selected studies and their relevance to granule and dosage-form performance. The selection of the studies was also based on the use of real-time monitoring technologies and modeling techniques; however, the absence of these elements was not considered a reason for exclusion. On the contrary, studies focusing solely on non-pharmaceutical applications or presenting a lack of significant methodological detail to establish relationships between formulation, process conditions, and product quality were excluded (Figure 2).

Each study was screened for key information, i.e., physicochemical and material properties of the APIs that included the type of granulation technique, the use of Process Analytical Technology, the modeling approach for data analysis, the critical process parameters under evaluation and the critical quality attributes that were tested in every case. These included, where applicable, granule particle size distribution, density, porosity, moisture content, flowability, and mechanical strength, as well as tablet properties such as hardness, friability, disintegration, dissolution, content uniformity, etc., as noted analytically per case in Table 2. Data from the selected studies were compiled in a comparative table (Table 2) and analyzed qualitatively to identify common trends, technological and formulation advances, and potential challenges and limitations. Furthermore, comparisons between PAT tools, taking into account the CQAs of the final formulation and including the properties of the APIs, are also evaluated and analyzed. This methodology enabled the selected literature to be evaluated not only according to the granulation technique employed but also according to the material and process factors governing its suitability and its impact on predefined product quality attributes.

Among the numerous examined publications found, studies were considered eligible to be included when they provided sufficiently detailed experimental or industrial evidence relevant to the objectives of this review and contributed to the coverage of the major granulation technologies and approaches identified in the literature. A total number of approximately 364 peer-reviewed studies were identified for the period 2020–2026, screened (by title/abstract) to exclude those meeting the excluding criteria, until the final 32 selected studies were found to meet necessary inclusion criteria and, most significantly, to offer significant evidence on the relationship between process parameters and product attributes. Particular consideration was given to studies providing quantitative experimental data, clearly defined process conditions, or documented applications of PAT/data modeling. Studies addressing technological or formulation advances, scale-up, continuous manufacturing, or emerging monitoring approaches were also considered eligible when they provided sufficient evidence to support meaningful comparison with established granulation processes, in both technological and methodological terms.

This review also has the following limitations. The literature search was conducted using a single database (PubMed) and the search was restricted to publications available in English, which may have resulted in the omission of some relevant studies. In addition, as this work was designed as a structured narrative review, no formal quality appraisal or risk-of-bias assessment was applied to the included studies. Consequently, the findings should be interpreted as a structured synthesis of the available literature rather than as a formal assessment of the strength or certainty of the evidence. Furthermore, differences among the selected studies in terms of formulations, experimental designs, process configurations, analytical methods, and reported performance metrics limited the possibility of direct quantitative comparison. Nevertheless, the review provides a structured overview of recent developments in pharmaceutical granulation and highlights relevant relationships among material attributes, process parameters, quality attributes, PAT approaches, and modeling strategies.

**Table 2:** Summary of selected case studies on pharmaceutical granulation processes, including applied PAT tools, investigated CPPs, evaluated CQAs/CMAs and modeling approaches.

Ref.	Scale	Method	PATs	CPPs	CQAs/CMAs	Data Modeling	Goal/Remarks
[25]	lab	Twin-Screw Wet Granulation (TSWG)	Near-Infrared Spectroscopy (NIR)	Solid feed rate	API concentration	Step test	Residence time distribution for process monitoring / Event propagation time of the response signal (EPT) 5–95%
[26]	industrial	Resonant Acoustic Wet Granulation (RAG)	-	Granulation liquid, L/S ratio	Cocrystal %, PSD, dissolution, tablet profile	Comparative analysis	Integrated RAG for cocrystal production / Co-crystal formation (>99%), granule size d50: >250 μm
[27]	industrial	TSWG	NIR	L/S ratio, screw speed/configuration, feed rate, drying	Moisture, PSD, flowability, tablet strength	Chemometric partial least squares (PLS)	Continuous glucose monohydrate production / PLS: error of calibration (RMSEC) 0.443, error of cross-validation (RMSECv) 0.531; Moisture accuracy detection with ± 0.5%
[28]	industrial	TSWG	NIR	Feed rate, screw speed/configuration, torque, shear force, temperature, water content, dryer parameters	LOD, PSD, tablet weight, hardness, disintegration, friability, thickness	D-optimal design, PLS, Principal Component Analysis (PCA)	Development and evaluation of a continuous production line / PLS: low prediction error with RMSEC_V_ is 1.12% for the tablet and 1.25% for granules
[29]	lab	TSWG	Thermal imaging camera and monitoring software	Production speed, granulator speed/temperature, blend composition	PSD, bulk and tapped density (BD & TD), tablet friability, strength, dissolution	-	Feasibility of potassium sodium tartrate tetrahydrate (PST) in a continuous line / 20-fold increase in productivity from 0.5 to 10 kg/h production rate
[30]	pilot	Fluid-bed Granulation (FBG)	-	Spray rate, atomization pressure, granulation binder	PSD, bulk density, flowability tablet weight, disintegration time, breaking resistance	2^3^ design, Students’ two tailed *t*-tests	Stable conversion of nanosuspension into tablets
[31]	pilot	Wet Granulation and Hot-Melt Extrusion (HME)	Flow property characterization of the granules via bulk and tapped density measurement by USP method 1	Melt-zone temperatures, print pressure and discharge, droplet aspect ratio and layer height	Angle of repose, granule flowability, BD &TD, dissolution	Mean Dissolution Time (MDT)	3D printing for tablet production by direct, continues granule feeding / Granule: Hausner ratio 1.08–1.15, i.e., flowable printable granules; Tablet: infill density indicated by a R^2^ value of 0.995
[32]	pilot	Dry Granulation	Piezoelectric force and chromatic confocal sensors	Feed rate, specific compaction force and speed, gap width, dwell time	Solid fraction (SF), elastic recovery	Mathematical–mechanical hybrid model	Hybrid model for solid fraction prediction / Prediction errors for the solid fraction < 2.3% for the roll compaction simulation
[33]	pilot	FBG	Confocal Raman microscopy	Polymer type, polymer/API ratio, viscosity, carrier dissolution rate, spray rate, atomization pressure, air flow and temperature	Redispersibility Index, PSD, nanoparticle layer structure, porosity, BD and TD	Comparative analysis	Parameters that affect the redispersion of API nanoparticles
[34]	lab	TSWG	Mixer torque rheometry	L/S ratio, MCC/mannitol ratio	API solubility, flowability, PSD, tensile strength (TS)	Multiple linear regression (MLR)	Predictive model for estimating optimal L/S ratio / Optimal at 75:25 MCC-mannitol ratio; optimum liquid amount was 2/3 of the L/S ratio at the maximum torque in the mixer torque rheometer
[35]	pilot	Twin-Screw Melt Granulation (TSMG)	-	Type, quantity and addition site of disintegrant, compaction force	PSD, BD, tablet weight, height, diameter, TS, SF, friability, disintegration, dissolution	Tablet mechanical analysis based on TS and SF	Effect of disintegrants on tablet profile
[36]	pilot	Dry Granulation	Artificial intelligence (AI) X-ray microscopy (XRM) image analysis tool	Roller compaction and tableting parameters	PSD	AI-based image segmentation, supervised machine learning, watershed algorithm	XRM model for granule microstructure monitoring
[37]	pilot	TSMG	-	Drug load, screw speed	Friability, PSD, tablet hardness, disintegration, dissolution	3 × 3 factorial design, MLR	Improvement of TSMG process for fenofibrate / Tablet: total drug release after 60 min was 14% in fast state simulated intestinal fluid and 42% in fast state and fed state simulated intestinal fluid
[38]	industrial	TSWG	-	Formulation, screw configuration, L/S ratio, mill screen size and type, residence time	Flowability, TS, PSD, API content	2 × 3 × 3 mixed-level design, full factorial 2^2^ design, linear regression	Robustness of a TSWG method / Use the midpoint between SP (starting point) and IP (inflection point) in mixer torque rheometer (MTR) curves to select L/S ratios
[39]	pilot	Wet Granulation	NIR	Spectral characteristics of components	Content uniformity, tablet color, shape, defects	Chemometric model, PCA and PLS	NIR/HPLC comparison for content uniformity monitoring / High analytical accuracy (RMSEP = 1.19%); meets content uniformity requirements (±15%)
[40]	pilot	High-Shear Wet Granulation (HSWG)	-	Binder type, binder/solvent concentration, sieve mesh	Dissolution, flowability, compressibility, plastic deformation, tablet hardness, TS	Multivariate physicomechanical and morphological compressibility analysis	Effect of HSWG on MCC compressibility / Ryshkewitch–Duckworth equations of MCC primary and secondary particles with R^2^ > 0.99
[41]	lab	HSWG/TSWG	Powder Rheometry and Torque monitoring	Impeller speed, granulation time, liquid feed time, screw speed, torque	PSD, BD, compressibility, flowability, dissolution, tablet weight, strength, dissolution, API content	Central composite design	Transition from HSWG to TSWG / Similar dissolution profiles (F2 greater than 50)
[42]	pilot	Dry Blending	Terahertz time-domain spectroscopy (THz-TDS)	Scattering, blend composition	PSD, BD & TD, homogeneity	Polynomial regression, Fourier transform, moving block standard deviation	THz-TDS as PAT for homogeneity and blending end-point / Low predictive errors of the content of 0.5–2.5%
[43]	pilot	TSWG	Particle size analyzer	L/S ratio, screw speed, powder feed rate	PSD	Long short-term memory (LSTM) network	Energy efficiency of TSWG using a LSTM model / 27% increase in capability
[44]	industrial	Fluid-bed Drying	-	Inlet air velocity and temperature	Moisture content, particle cohesion force	Coupled computational fluid dynamics (CFD)–discrete element method (DEM)-based model	Modeling of granule drying process dynamics / Increase in drying rate with the increase in inlet air velocity and inlet air temperature
[45]	pilot	Dry Granulation	-	Roll force and gap	True density, compression profile, frictional properties, PSD, flowability, API content, SF	Mathematical model based on Johansons’ theory	Material profiling and roller compaction modeling for optimal API consumption
[46]	industrial	TSWG	-	Screw speed, material throughput, L/S ratio	PSD	Kernel Mean Embedding (KME)	KME model for the prediction of PSD / Low average RMSE value of 0.0876
[47]	industrial	FBG	NIR and particle size analyzer	Airflow rate, atomization pressure, runtime, air temperature/pressure, relative/absolute humidity	PSD, granule moisture	PLS and PCA	NIR-parameters model for moisture and PSD prediction / 2.7% rel. diff. (RMSEP) in favor of combined NIR + particle size model, i.e., improves granule size prediction, compared to the particle size -only model
[48]	lab	TSWG	Spatial Filter Velocimetry (SFV) & Dynamic Image Analysis (DIA)	L/S ratio, mill type, screen, intensity	PSD, flowability	Deterministic image-processing pipeline, one-factor-at-a-time process mapping	DIA for in-line characterization of PSD / Use of vibratory feeder with periodically altered feeding intensity to temporarily reduce the mass flow of the material passing in front of the camera, resulting in higher imaging analysis
[49]	pilot	HSWG	-	L/S ratio, impeller speed, granulator scale	Granule size G50	Multivariate analysis, PLS, PCA	Granule size prediction model based on the literature and experimental data / Larger L/S ratios were responsible for larger granule sizes; average absolute prediction error at 26.3 μm
[50]	pilot	TSWG	NIR	L/S ratio, drying time	Moisture content, tablet TS, porosity, compressibility, compactability	Reynolds-type, power-law porosity model	Tablet profiling using an L/S and moisture-based model / Model RMSE of 0.17 tablet tensile strength (MPa) allowing more flexibility
[51]	industrial	HSWG	-	Impeller force, liquid flow, material source, drying time and temperature, air flow	PSD, tablet TS, MCC particle size, product temperature	Ridge regression, random forest model	Machine learning for PSD and tensile strength prediction / Screening-type-approach to utilize the machine learning models
[52]	pilot	FBG	Hyperspectral Imaging (HSI)	-	Moisture content, PSD, compound content	Deep residual network algorithm, PCA and PLS	HSI-ResNet model for real time CQA prediction / Satisfactory predicting values R^2^ > 0.9 and small root mean square error
[53]	pilot	TSWG	Infrared camera	Mass feed rate, L/S ratio	PSD, API content, granule temperature	Energy balance and in-line thermal imaging	Granule wetting efficiency in a TSWG process
[54]	industrial	HSWG	NIR	Water and Na_2_CO_3_ quantity, impeller and chopper speed, wetting time, pump speed, drying air temperature	Tablet dissolution rate	PLS	NIRS for monitoring ibuprofen sodium formation / Models with accuracy and precision R^2^, Q^2^ > 0.9
[55]	pilot	HSWG	NIR	Time, adding water amount (mL), stirring (rpm), chopper (rpm)	PSD, tapped density, Hauser ratio	Partial least squares regression analysis (PLSR)	NIRS/PLSR model for real time CQA monitoring / ModelESS is the fitting index of the model
[56]	pilot	FBG	Off-line and at-line NIR, at-line Raman	Component, Granulation Liquid [*w*/*w*%], Granulate [*w*/*w*%]	API content	PLS	Quantifying API content during amorphous solid dispersions preparation / Multiple PLS models tailored to narrower concentration ranges needed

## 3. Results

### 3.1. Granulation Techniques and Process Configurations

Across the multitude of available techniques, a clear preference emerges towards the use of wet granulation and especially twin-screw wet granulation (TSWG) and high-shear wet granulation (HSWG). This represents the tendency of the pharmaceutical industry to demand more robust and easily scalable configurations, that have been thoroughly tested and extensively evaluated. Particularly, TSWG has gained significant attention as it has demonstrated its compatibility with various granulation-to-tableting production lines. An easily customizable technique, specifically fitted for different product needs, can be seamlessly integrated into configurations that combine blending, granulation, drying, milling and tableting. Introducing TSWG to industrial-scale pharmaceutical production is a step towards enhancing process efficiency, minimizing material loss and monitoring product quality in real-time [25,27,41].

**Figure 3:**
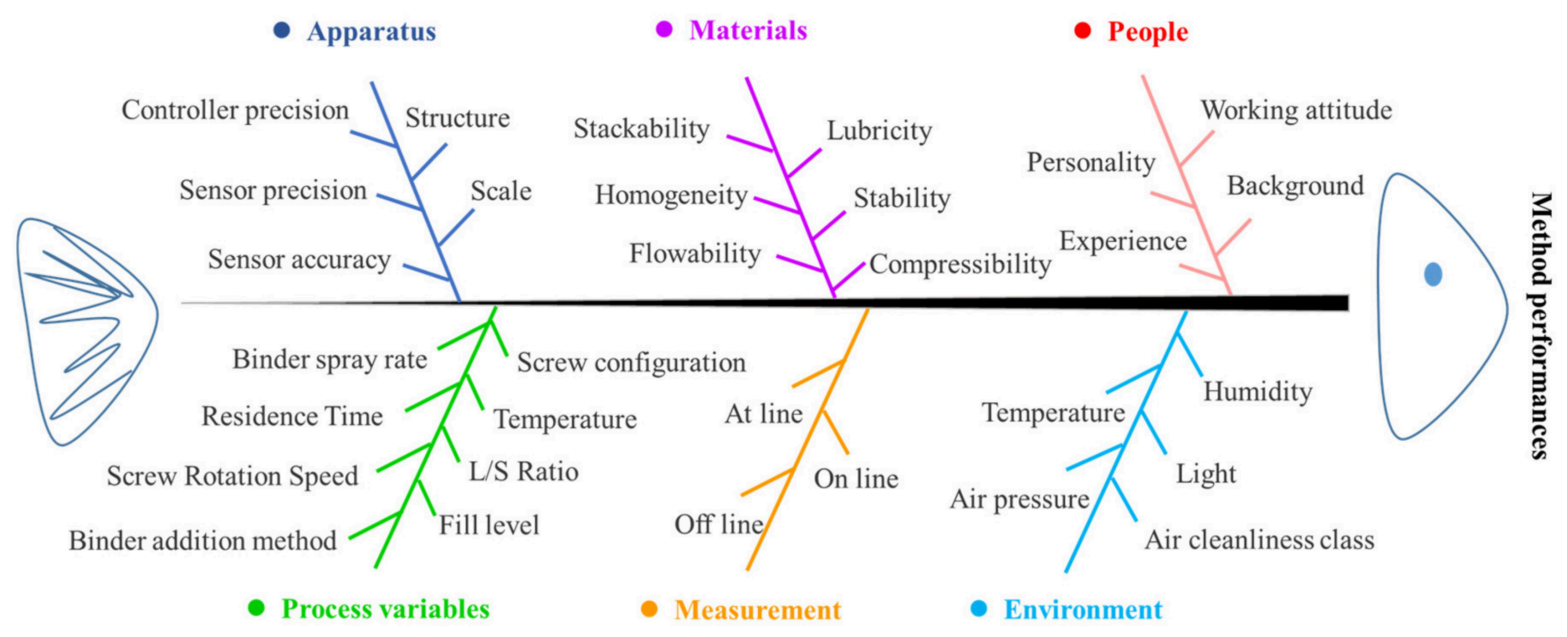
Ishikawa diagram for the twin-screw granulation process behavior. Adapted by Zhao et al. [57], with Creative Commons CC BY 4.0 open-access license.

Considering the different factors affecting the process behavior TSWG system (Figure 3), a systematic understanding of the dynamic change in the characteristics of the material is required, during granulation process, via modeling the process through statistical, mechanistic, and hybrid models. The process design, control, and variable optimization of the TSWG process can also be developed by modeling techniques [57].

The quality of granules produced by TSMG is strongly influenced by critical process parameters, including barrel temperature, screw speed, feed rate, screw configuration, and binder concentration. These parameters determine the residence time, shear stress, and degree of binder distribution within the powder bed, ultimately affecting granule growth and product performance. Owing to its continuous nature, TSMG is well suited for integration into continuous melt granulation manufacturing lines and can readily incorporate PAT tools, including near-infrared (NIR) spectroscopy, Raman spectroscopy, and torque monitoring, to enable real-time process monitoring and support real-time release testing. Consequently, TSMG has attracted increasing interest as a robust and efficient platform for the manufacture of immediate- and modified-release solid dosage forms, as well as for formulations containing poorly water-soluble compounds or high drug-loads. Some challenges remain, however, in understanding the complex interactions between formulation composition, screw design, shear history, and thermal exposure [12,13,58,59].

High-shear wet granulation (HSWG) is the most widely used batch granulation technique in the pharmaceutical industry due to its robustness, versatility, and ability to produce granules with excellent flowability and compressibility. The process is typically performed in a high-shear mixer equipped with an impeller and a chopper, where powder particles are rapidly mixed while a granulating liquid is sprayed or added into the powder bed. The impeller provides intensive mixing and densification of the powder, whereas the chopper breaks up oversized agglomerates and promotes the formation of a narrow granule size distribution. Granule formation occurs through successive wetting, nucleation, growth, consolidation, and breakage mechanisms, with the balance between these phenomena determining the final granule characteristics. Critical formulation variables include API particle size, excipient properties, binder type and concentration, and liquid-to-solid ratio, while critical process parameters such as impeller speed, chopper speed, granulation time, binder addition rate, and endpoint determination significantly influence granule size distribution, porosity, density, friability, and tabletability. Although HSWG consistently produces high-quality granules with excellent content uniformity and is particularly suitable for low-dose formulations, cohesive powders, and poorly flowing materials, it remains an energy-intensive process because of the subsequent drying step and may not be appropriate for moisture- or heat-sensitive APIs. PAT techniques, referred also in Table 2 and power consumption analysis have been investigated for real-time granulation endpoint detection and process control. In addition, computational approaches, referred also in Table 2, are increasingly being used to investigate particle interactions, granule growth mechanisms, and scale-up behavior. Owing to its long history of industrial implementation, well-established regulatory acceptance, and extensive process knowledge, HSWG continues to serve as the benchmark against which emerging continuous granulation technologies, including twin-screw wet granulation, are evaluated [60,61].

Going a step further, alternative and emerging wet granulation configurations have been developed to tackle certain processing constraints. Resonant acoustic granulation (RAG) represents an alternative wet-granulation approach, including approaches involving granulation liquids, that can provide enhanced process flexibility [26], while twin-screw melt granulation (TSMG) enables agglomeration using a thermoplastic binder without the need for an aqueous granulation liquid [35]. Plus, liquid-free granulation approaches have also been explored, as reported by Fekete et al. [29]. These approaches have been introduced to enhance energy efficiency and widen the applicability of continuous granulation processes, yet they remain less established, requiring further investigation into their scalability and regulatory acceptance. As pharmaceutical manufacturing shifts to integrated and continuous configurations, the need for reliable process monitoring continues to increase. The real-time quality control of in-line, on-line and at-line analytical tools, positions Process Analytical Technologies at the forefront of granulation monitoring.

### 3.2. Implementation of Process Analytical Technologies (PATs)

**Figure 4:**
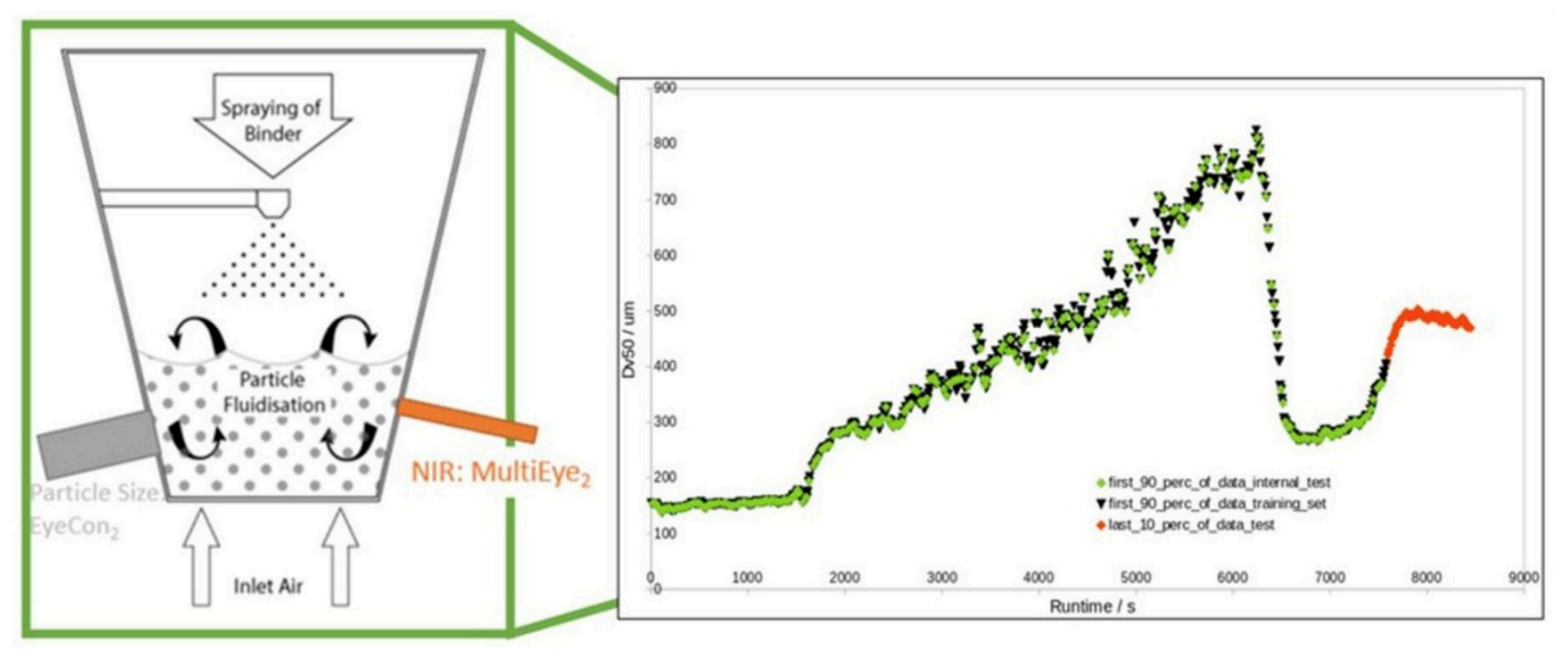
Schematic diagram of the fluidized bed spray granulation process, with an NIR and online particle size measurement system. Adapted by [47], with Creative Commons CC BY 4.0 open-access license.

Traditionally, drug quality has been assessed at the end-point of each production step, underlining that batch-processing dictates current pharmaceutical principles. In recent years, research has developed a dynamic towards implementing process analyzers, striving to uncover novel, real-time techniques that enable better understanding of product behavior during granulation. PAT was introduced by regulatory agencies as a framework for designing, analyzing, and controlling manufacturing processes through timely measurements of critical quality and performance attributes. Among these, spectroscopic methods like Near-Infrared spectroscopy and Raman spectroscopy have emerged as leading PAT tools. These techniques are frequently employed for in-line or on-line granule and tablet monitoring, targeting key product attributes like API concentration, particle size distribution, moisture and content uniformity. Because moisture plays a critical role in granule formation, NIR measurements are often integrated into feedback control systems to maintain consistent process conditions (Figure 4), while Raman measurements are particularly useful when monitoring formulations containing polymorphic materials or multiple active ingredients. By offering non-destructive rapid data acquisition when fitted to continuous production lines, spectroscopic analyzers showcase significant applicability in both experimental and industrial settings [25,27,28,33,39,47,54,55,56].

Near-infrared (NIR) spectroscopy provides rapid, non-destructive, and in-line measurements of moisture content, blend uniformity, API concentration, and granule density. Its high acquisition speed and compatibility with continuous manufacturing make NIR particularly suitable for monitoring wet granulation processes; however, its broad and overlapping spectral bands often require advanced chemometric modeling for accurate quantitative analysis. Raman spectroscopy provides higher chemical specificity than NIR and is less sensitive to water interference, making it especially valuable for identifying polymorphic transformations, monitoring API distribution, and analyzing multicomponent formulations. Nevertheless, Raman measurements can be affected by fluorescence, limited sampling volume, and relatively high instrumentation costs. Thermal imaging has recently emerged as a complementary PAT technique capable of monitoring temperature distributions across the powder bed or granulation equipment. Because temperature strongly influences binder melting, solvent evaporation, and granule growth, thermal imaging enables the identification of process heterogeneities, overheating, and equipment malfunctions in real time. However, it primarily provides indirect information on product quality and is generally used alongside spectroscopic methods rather than as a standalone monitoring tool. Image-based analytical techniques, including dynamic image analysis, high-speed imaging, and machine vision systems, provide direct measurements of granule size, shape, morphology, and growth kinetics, thereby offering valuable insight into the physical mechanisms of nucleation, coalescence, consolidation, and breakage. Despite these advantages, image analysis remains challenging under industrial conditions because of particle overlapping, dust generation, variable illumination, and the computational complexity associated with processing large image datasets [25,27,28,33,39,47,54,55,56].

**Figure 5:**
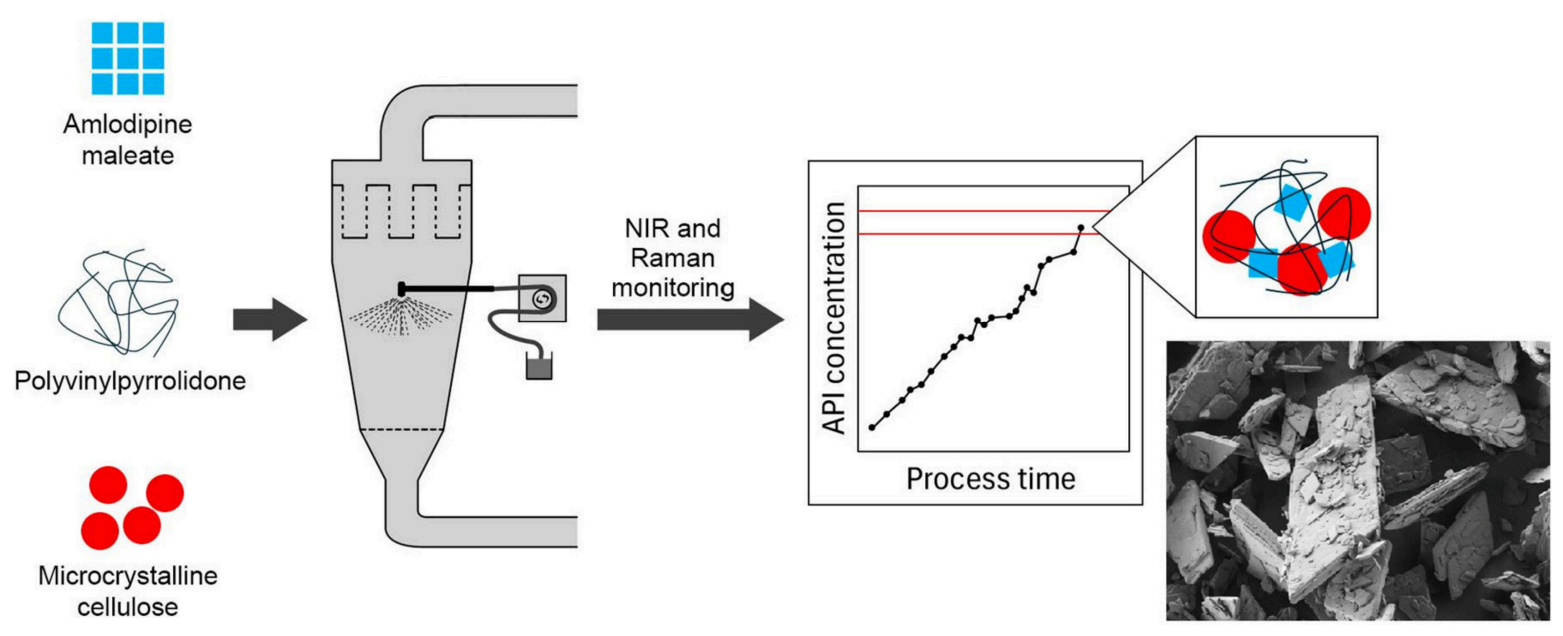
Quantification of amlodipine maleate content in amorphous solid dispersions produced by fluidized bed granulation using process analytical technology tools along with illustration techniques (SEM microscopy). Adapted from [56], with Creative Commons CC BY 4.0 open-access license.

Complementary to spectroscopic PATs, research has explored the use of dynamic imaging techniques for characterization of granule formation and growth, providing information on granule size, shape, and morphology. Approaching granulation with the use of image-based techniques offers a direct insight into particle dynamics that are not entirely depicted in spectroscopic monitoring alone (Figure 5). Consequently, no single PAT technique is capable of comprehensively characterizing all aspects of the granulation process. Increasingly, pharmaceutical manufacturers are adopting multimodal PAT strategies that combine complementary analytical techniques with chemometric models and advanced process control systems. Such integrated approaches improve process understanding, enhance predictive capability, and support Quality-by-Design (QbD) and real-time release testing (RTRT) initiatives within modern continuous pharmaceutical manufacturing [62].

Knowledge of the mechanisms of granule nucleation, coalescence, breakage and attrition uncovers valuable information and leads to better process understanding. However, systematic implementation of imaging techniques remains challenging due to high data noise levels and difficulty in industrial-scale integration [29,48,53]. Effective monitoring of granulation techniques requires not only robust and sensitive tools, but also a comprehensive understanding of the influence that process parameters have on pharmaceutical product quality.

The applicability of PAT techniques depends on the manufacturing technology, material properties, and quality attributes requiring monitoring. In high-shear and fluid-bed wet granulation, NIR spectroscopy can monitor moisture and chemical composition, while temperature, airflow, torque, and pressure measurements provide information on process conditions and granulation progression. Imaging and particle-size analysis can characterize granule size and morphology. For roller compaction, process measurements such as compaction pressure, roll gap, roll speed, and feed rate can be combined with NIR or Raman spectroscopy to monitor material composition and moisture, while ribbon density, strength, and granule size provide intermediate-product information. In melt granulation using twin-screw extrusion, temperature, screw speed, feed rate, and torque can be monitored alongside NIR or Raman measurements to assess chemical composition and solid-state changes. Imaging can further characterize granule or extrudate properties. Importantly, PAT measurements do not necessarily measure CQAs directly; correlations with CQAs may require calibration, chemometric, or process models. Integrating complementary PAT tools can therefore improve process understanding, monitoring, and control and support QbD and, where validated, real-time release testing.

A summarizing comparative table of the PAT techniques mentioned above is presented in Table 3, each one of them offering distinct advantages depending on the process requirements and the quality attributes of interest.

**Table 3:** Comparative table of the PAT tools referred to in [25,26,27,28,29,30,31,32,33,34,35,36,37,38,39,40,41,42,43,44,45,46,47,48,49,50,51,52,53,54,55,56].

PAT Technique	Measures	Advantages	Limitations	Typical Granulation Applications
**NIR spectroscopy**	Moisture, API concentration, blend uniformity	Fast, non-destructive, suitable for in-line monitoring	Overlapping spectra, requires chemometric models	Wet granulation endpoint detection, moisture control
**Raman spectroscopy**	Chemical composition, polymorphism, API distribution	High chemical specificity, low water interference	Fluorescence, higher cost, smaller sampling volume	API distribution, polymorph screening, content uniformity
**Thermal imaging**	Surface temperature, heat distribution	Non-contact, detects thermal heterogeneity	Indirect quality measurements, surface only	Melt granulation, drying, equipment monitoring
**Image analysis/Machine vision**	Particle size, shape, morphology, granule growth	Direct visualization of granule evolution	Sensitive to dust, lighting, particle overlap, computationally intensive	Granule growth, size distribution, process development
**Acoustic emission/Torque/Power consumption**	Mechanical behavior during granulation	Simple integration, low cost, endpoint detection	Indirect measurements	High-shear granulation endpoint determination

### 3.3. Critical Process Parameters (CPPs) Across Granulation Methods

A thorough examination of the available literature reveals trends between certain granulation processes and the critical parameters that govern their central mechanisms. Minor or major changes in these parameters can greatly alter the granulation continuum causing shifts in product throughput or affecting overall quality.

One of the better understood and extensively investigated relationships between a granulation method and a CPP is that of the liquid-to-solid (L/S) ratio in TSWG. In wet granulation techniques, addition of a liquid binder is essential for the formation of intra-particle bridges between APIs and excipients, leading to granule growth. Inadequate binder addition can lead to insufficient granulation and excessive particle breakage resulting in poor cohesion and content uniformity. On the contrary, excessive liquid introduction is a frequent cause behind over-wetted granules, leading to slurries and subsequent product rejection. The effect of L/S ratio in TSWG has been deemed of great importance, viewed as the primary process parameter affecting particle size distribution, flowability and tableting properties, like table strength [27,34,38].

Following L/S ratio, screw speed constitutes a defining critical parameter in a TSWG process. Throughout the relevant research, screw speed has been shown to influence the granule residence time distribution (RTD), the applied shear-force intensity, as well as the binder dispersion inside the formulation. High screw speeds lead to a smaller RTD and more intense shear forces, while low screw speeds favor granule growth but cause content heterogeneity. Alterations in screw speed have also been linked to changes in major granule quality attributes including PSD, granule density, porosity and mechanical strength. Consequently, screw speed is not an entirely independent parameter, as it functions in synergy with other major TSWG factors such as L/S ratio, solid feed rate and screw configuration [27,28,38,43,46].

Beyond method-specific parameters, a common theme arises across recent studies, centering around CPPs dependent on process configuration. Rather than acting as separate factors, CPPs are increasingly operated in synergy as complementary variables whose total effect dictates the outcome of a granulation process. This is a major reason behind their monitoring in continuous production lines, where slight deviations in input parameters can cause a cascade of unwanted effects in downstream processing. PATs come to complement the identification and management of CPPs, aiding at the reinforcement of QbD principles and the stability of CQAs.

### 3.4. Critical Quality Attributes (CQAs)

To maintain granule and subsequent tablet and capsule quality, a plethora of characteristics need to be evaluated across all production stages. The term CQAs encompasses all physical, morphological and physicochemical traits that could potentially have a major impact on product quality and safety. Similar to CPPs, critical quality attributes constitute multidimensional entities that function not individually but rather represent an interconnected and dynamic network based on the principles of cause and effect.

Among the various CQAs, investigated in recent years, structural and physical attributes emerge as the most influential. Particle size distribution (PSD) remains the reigning metric for granulation characterization, providing insight into the effectiveness of the used agglomeration method [36,42,43,46]. A monomodal distribution, with a narrow and controlled particle size offers optimal flowability and structural integrity in produced granules. However, studies highlight that PSD is not sufficient by itself to fully characterize granule quality. Other attributes like density and porosity are assessed at the same time, as they provide a more holistic approach for granule characterization [29,30,33].

Other than the structural and physical attributes, great care is taken to evaluate the mechanical properties of granules and the impact that they have on the final performance of solid dosage forms. Frequent indicators for granule stability against mechanical stress are friability and tensile strength (TS) after compression. The mishandling of process parameters during production, can lead to excessive fine generation, poor content uniformity and improper particle agglomeration, hinders the aforementioned indicators resulting in underperformed tabletability. Consequently, emphasis in the modern literature is placed on the simultaneous evaluation of mechanical granule and tablet attributes to establish robust relationships between granulation and final product quality [28,29,35,37].

Another category of CQAs that has gained momentum in process monitoring, particularly in continuous production, is content-related characteristics. Proper dosage is inextricably linked to a stable API concentration and overall content uniformity, especially in continuous processes where the dynamic landscape of the method presents several challenges. Evidently, relying solely on endpoint testing is not enough for the proper monitoring of these quality attributes [25,38,39,45,53].

Finally, functional CQAs including dissolution, redispersibility and moisture extend the concept of product quality even further, especially in formulations containing poorly soluble or nano-dispersed APIs where slight deviations can greatly alter the release profile of an active ingredient. This is a clear modern pharmaceutical development effort to shift the focus towards more inclusive performance-oriented production, providing a link between granulation and bioavailability outcomes [44,50,52,54].

### 3.5. Data Modeling and Advanced Analytical Approaches

Developing new tools for monitoring process parameters in granulation generates a wide range of data that needs to be evaluated, in order to extract meaningful information. Data analysis leads to a better understanding of granulation and to the possible development of predictors for monitoring changes in CPPs, supporting real-time process monitoring and control. Both empirical and mechanistic, as well as hybrid, models have been implemented in CPP analysis, using mathematical approaches and physics-based principles respectively to reveal intrinsic relationships between process parameters and product quality.

**Figure 6:**
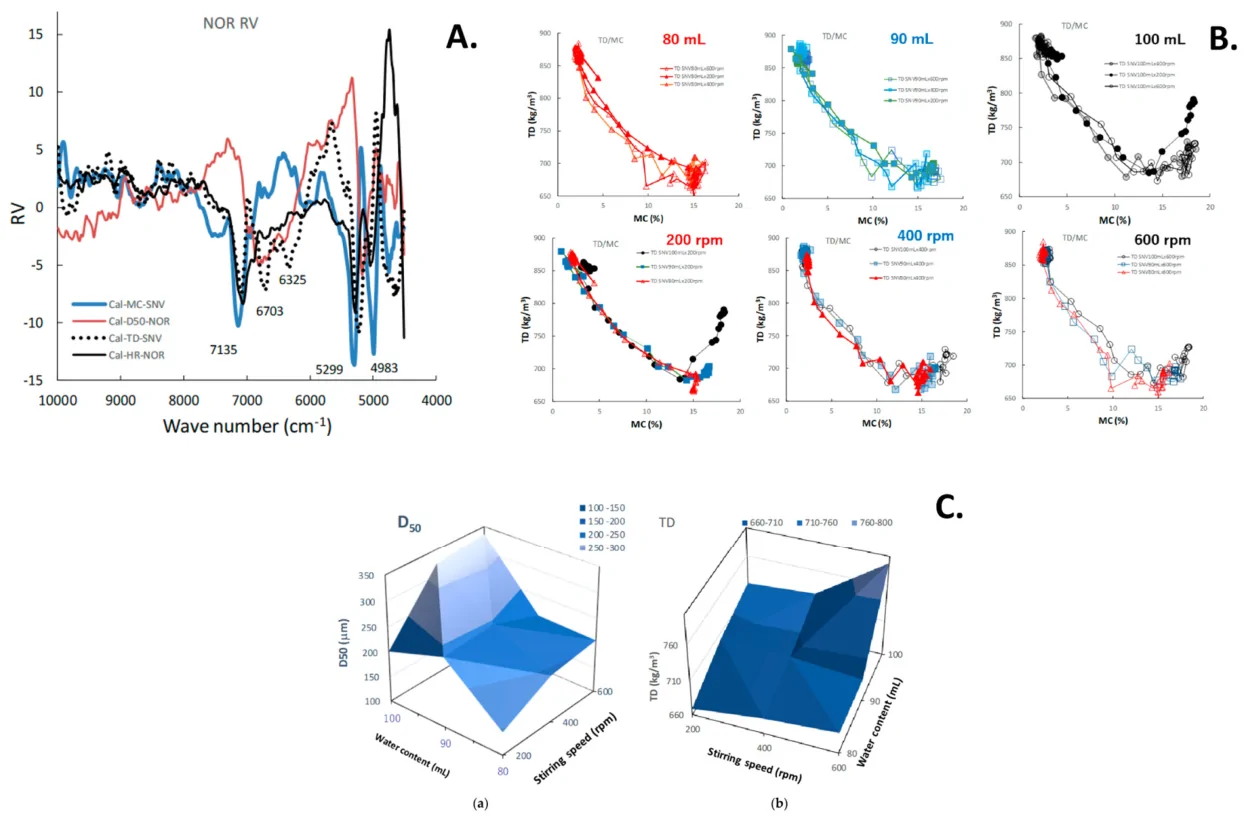
Development of a NIRS/PLSR model for real-time CQA monitoring: (**A**) Calibration models for predicting the MC, D50, TD, and HR during the HSWG process. (**B**) The effect of CPPs on the real-time CQA profiles (of D50 (**a**) and TD (**b**)) of the HSWG granules. (**C**). Three-dimensional plots of pharmaceutical properties of the final HSWG products predicted using the NIR/PLSR method versus their critical process parameters. Adapted by [55], with Creative Commons CC BY 4.0 open-access license.

Both experimental and industrial environments are fully compatible with empirical modeling approaches, making them the preferred method for data processing and evaluation as seen throughout the literature. The purely statistical identification of relationships between CPPs and CQAs according to the principles of DoE offers a framework for systematic exploration of the process space and operating regions, highlighting granulation effects and interactions [32,37,40,45]. On the other hand, multivariate data analysis through methods such as Principal Component Analysis (PCA) and partial least squares (PLS) are frequently implemented for data extraction and processing in cases of PAT use. These methods often lead to the development of predictive models for efficient performance monitoring, early deviation detection and real-time quality control (Figure 6). These chemometric methods facilitate dimensionality reduction, variable selection, process classification, and the development of predictive models for moisture content, particle size distribution, granule density, and API concentration. Consequently, empirical models have become indispensable for real-time process monitoring, early fault detection, and real-time release testing (RTRT), particularly within Quality-by-Design (QbD) frameworks [27,28,39,47,49,55]. Empirical models remain the most widely adopted modeling approach in pharmaceutical granulation because they can efficiently describe relationships between CPPs and CQAs using experimental data. Statistical methodologies based on Design of Experiments (DoE), response surface modeling and multivariate regression facilitate process optimization and formulation development while requiring relatively low computational effort [45,46,50].

**Figure 7:**
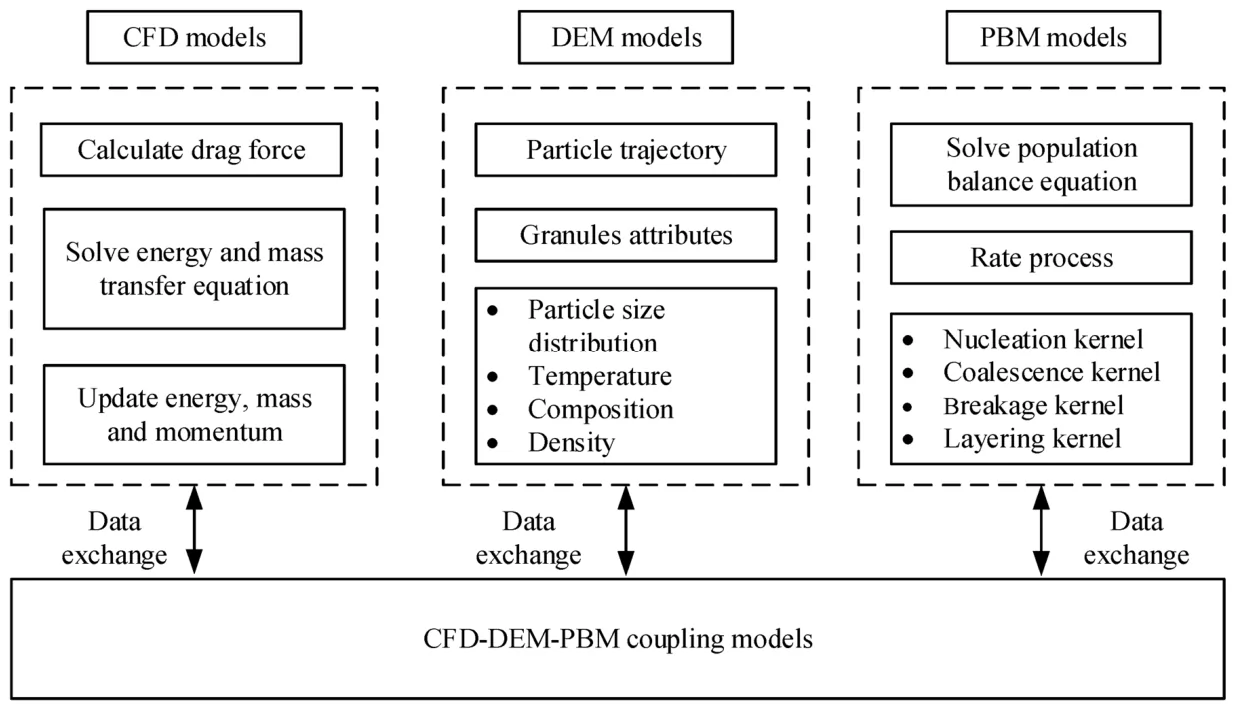
Schematic of the models for granulation. Adapted by Zhao et al. [57], with Creative Commons CC BY 4.0 open-access license.

Contrary to statistical models, mechanistic approaches aim to describe granulation methods based on physics principles and particle system dynamics, governing particle interactions and transport phenomena. These models improve scientific understanding of granule formation by explicitly representing mechanisms such as wetting, nucleation, coalescence, consolidation, breakage, and attrition. Across the available research, models like the population balance model (PBM), discrete element method (DEM) and computational fluid dynamics (CFD) that describe mass and energy transfer, particle interactions and agglomeration mechanisms offer valuable information concerning varying operating conditions (Figure 7). For example, the discrete element method (DEM) simulates individual particle motion and contact mechanics, providing detailed insight into particle collisions, mixing behavior, and force distributions within granulation equipment. Computational fluid dynamics (CFD) complements these approaches by modeling fluid flow, heat transfer, and mass transport, thereby enabling the investigation of binder distribution, drying phenomena, and thermal gradients during granulation. Mechanistic models based on CFD, DEM and PBM describe particle interactions, heat and mass transfer, and granule growth mechanisms, providing detailed insight into granulation behavior under varying operating conditions. Coupled CFD–DEM models have recently demonstrated significant capability for simulating pharmaceutical drying processes and improving process understanding during scale-up [44].

These models provide valuable insights into granule size distribution dynamics and are frequently used for process optimization and scale-up studies. While mechanistic models provide a thorough understanding of complex and multidimensional mechanisms, the associated computational burden constitutes their broader industrial use as a challenge. Together, these models provide valuable information for process optimization, equipment design, scale-up, and technology transfer by improving understanding of the influence of operating conditions on final product quality. Nevertheless, they remain essential tools for process understanding and interpretation of observed trends during granulation [32,40,44].

Although mechanistic models provide considerably greater process insight than purely statistical approaches, their implementation is often constrained by high computational demands, extensive parameter estimation, and the need for detailed material property data. Consequently, they remain more commonly employed during process development and scientific investigations than in routine industrial manufacturing. Recent advances in computational power, however, have facilitated the development of coupled modeling strategies that integrate CFD, DEM, and PBM methodologies. These hybrid mechanistic frameworks simultaneously capture particle-scale interactions and process-scale transport phenomena, resulting in improved predictive capability and more realistic simulations of complex granulation systems. Furthermore, modeling entire pharmaceutical manufacturing lines requires individual unit-operation models to be parameterized and subsequently linked into integrated flowsheet simulations, allowing information to be transferred between upstream and downstream processes and enabling holistic process optimization [16].

**Figure 8:**
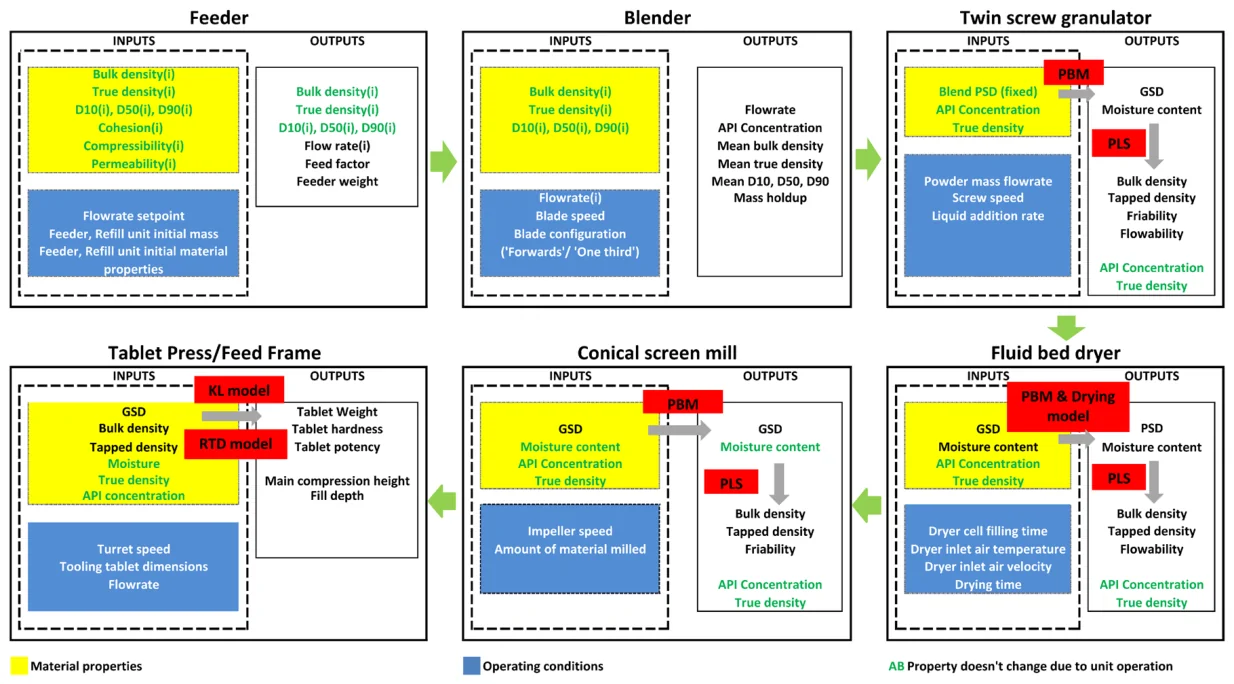
Schematic representation of various modeling approaches within flowsheet model for tablet manufacturing by wet granulation. Adapted from Metta et al. [16], with Creative Commons CC BY 4.0 open-access license.

Hybrid approaches combining CFD, DEM, and PBM methodologies have emerged as powerful tools capable of capturing both particle-scale interactions and process-scale behavior. Such integrated models, providing enhanced predictive capability and support rational process design, integrating mechanistic knowledge with experimental databases and data fusion techniques, have been proposed to improve prediction accuracy, while reducing computational complexity and data requirements. Such approaches are particularly attractive for pharmaceutical manufacturing because they can leverage existing process knowledge while continuously updating predictions using real-time PAT measurements. Hybrid modeling also provides the foundation for digital twin technology, in which a virtual representation of the manufacturing process is continuously synchronized with sensor data to monitor process performance, predict future operating conditions, evaluate process deviations, and optimize control strategies without interrupting production. Moreover, towards the modeling of the whole process, each individual unit operation may need a different model to be developed and parameterized, until a flowsheet model (Figure 8) is built via connecting the inlet of a unit to the outlet of the preceding unit, wherein the different modeling approaches of each unit interact, towards the smooth information transfer [14,49,50].

Beyond purely statistical and mechanistic models, hybrid modeling and advanced system exploratory frameworks support the efficient integration of process knowledge with real-time data. Artificial intelligence (AI) and machine learning are being evaluated for their applicability in granulation processes, introducing a new and dynamic frontier for modeling granulation procedures. Machine learning algorithms, including random forests, artificial neural networks, support vector machines, and gradient boosting methods, have demonstrated significant potential for predicting CQAs and enabling proactive process control [36,43,51,52].

An emerging area of pharmaceutical process modeling is the development of digital twins (DTs), which represent dynamic virtual replicas of physical manufacturing processes that are continuously updated using real-time PAT measurements and process data. Unlike conventional simulation models, digital twins integrate first-principles (mechanistic) models, empirical relationships, and data-driven algorithms to mirror the behavior of the physical process throughout its lifecycle. Within pharmaceutical granulation, digital twins enable continuous monitoring of CPPs and CQAs, allowing operators to predict process evolution, evaluate the impact of parameter changes, and identify potential deviations before product quality is compromised. This capability supports virtual experimentation, process optimization, and risk assessment while reducing the need for extensive experimental trials and facilitating technology transfer and scale-up. Coupled with Model Predictive Control (MPC), digital twins can move beyond process monitoring towards autonomous process control. MPC employs dynamic process models to predict future process behavior over a defined time horizon and determines optimal control actions while considering operational constraints. By continuously updating model predictions with real-time PAT measurements, MPC can automatically adjust operating conditions such as liquid addition rate, screw speed, barrel temperature, impeller speed, or feed rate to maintain predefined CQAs despite process disturbances. Such adaptive control strategies have demonstrated significant potential for improving process robustness, reducing material waste, and enabling real-time release testing (RTRT). As pharmaceutical manufacturing continues to transition towards continuous production and Industry 4.0, the integration of digital twins, MPC, PAT, and artificial intelligence is expected to provide the foundation for intelligent, self-optimizing manufacturing systems capable of achieving enhanced product quality, greater manufacturing flexibility, and improved regulatory compliance [63,64,65,66].

Artificial intelligence (AI) and machine learning (ML) have further expanded the capabilities of pharmaceutical process modeling and represent an important component of Industry 4.0 manufacturing. Unlike conventional regression methods, machine learning algorithms are capable of identifying complex nonlinear relationships between process variables and product quality, within large, multidimensional datasets generated during granulation. Algorithms including artificial neural networks (ANNs), random forests (RF), support vector machines (SVMs), gradient boosting methods, Gaussian process regression, and deep learning architectures have demonstrated considerable potential for predicting CQAs, identifying process deviations, optimizing operating conditions, and supporting autonomous process control [36,43,51,52]. For example, artificial neural networks, random forests, gradient boosting algorithms and deep learning architectures have been successfully applied for predicting particle size distribution, tablet tensile strength, moisture content and energy consumption during pharmaceutical granulation [43,51,52]. Combined with PAT and advanced control systems, these approaches enable predictive maintenance, adaptive process optimization, and enhanced decision-making throughout pharmaceutical manufacturing.

Another rapidly evolving area is Explainable Artificial Intelligence (XAI). Although conventional machine learning algorithms often provide highly accurate predictions, many operate as “black-box” models, limiting their acceptance in highly regulated pharmaceutical environments. Explainable AI seeks to improve model transparency by identifying which process variables contribute most significantly to model predictions. Techniques such as SHapley Additive exPlanations (SHAPs), Local Interpretable Model-Agnostic Explanations (LIMEs), feature importance analysis, and attention-based neural networks enable greater interpretability while maintaining predictive accuracy. Increased model transparency improves regulatory confidence, facilitates model validation, and supports Quality-by-Design (QbD) principles by enhancing scientific understanding of process behavior [67].

These developments are closely linked to real-time release testing (RTRT), one of the primary objectives of Process Analytical Technology. Rather than relying solely on end-product quality testing, RTRT enables product release based on continuous monitoring of CQAs throughout manufacturing. Advanced predictive models, supported by PAT measurements and machine learning algorithms, provide continuous estimates of product quality, thereby reducing manufacturing cycle times, minimizing waste, and improving production efficiency while ensuring regulatory compliance. A comparative table (Table 4) of the modeling approaches described above summarises their addvantages and respective limitations.

## 4. Discussion

### 4.1. Key Findings and Current State of Pharmaceutical Granulation

**Figure 9:**
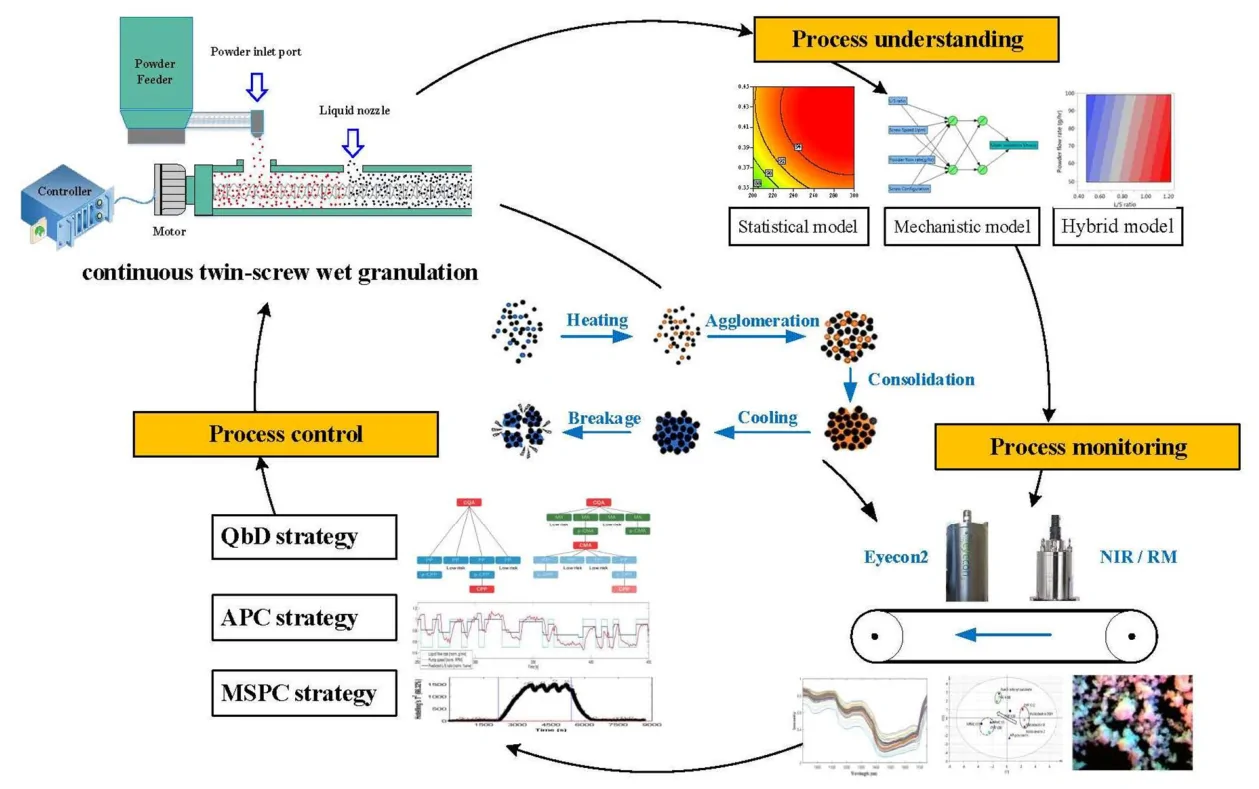
The complementary utilization of process modeling, monitoring with PATs control strategies within TSWG process towards product quality optimization. Adapted from Zhao et al. [57], with Creative Commons CC BY 4.0 open-access license.

The main objective of the present review was to uncover modern patterns and trends in the granulation process of solid dosage forms. Throughout the evaluated research a clear shift from traditional batch processing towards continuous manufacturing can be noticed. While conventional batch processing remains widely implemented, future pharmaceutical production is expected to become increasingly automated, interconnected, and knowledge-driven through the adoption of Pharma 4.0 principles. This is an indication that the pharmaceutical world follows the paradigm of the food and petrol industries, that have already embraced the philosophy of continuous and uninterrupted production. This shift creates a direct need for a total remodel of in-process control, revealing that end-point testing is not merely enough for monitoring continuous production lines and that novel real-time analytical tools need to be utilized. Granulation is not an isolated unit operation anymore but is treated as a dynamic system encompassing relationships between CPPs and CQAs, monitored and evaluated using PATs and advanced modeling techniques. This widely used method is steadily evolving into a data-rich and interconnected process, within an environment driven by QbD (Figure 9).

To support the constantly maturing granulation landscape, both established and developing technologies are researched throughout the available literature. The prevalence of twin-screw granulation is not a random occurrence meant exclusively for experimentation. This dynamic technology offers unparalleled flexibility for effective use in continuous production lines, while representing a great platform for efficient control of CPPs. The use of process parameters as key functional indicators has also shifted, with the focus being on uncovering synergistic behaviors of multiple parameters rather than relying on single one-at-time factors. A similar transition occurs relative to the evaluation of product quality through CQAs. Even though granule characteristics such as PSD and content uniformity are of paramount importance, modern research strives to create a multi-dimensional quality approach, based on a holistic picture of particle structure, mechanics and overall function. In this way, CPPs and CQAs operate as a causal network, uncovering intricate relationships, rather than acting as independent variables.

Widespread implementation of Process Analytical Technologies has shifted the traditionally static quality assessment procedures towards more dynamic real-time and continuous data acquisition processes. By reshaping the understanding of fundamental pharmaceutical granulation principles, PATs enable efficient CQAs monitoring by interpreting the propagation of CPPs across the production of solid dosage forms. The use of non-destructive, easily adaptable spectroscopic techniques has proven a highly effective PAT category, especially when it comes to monitoring moisture, API concentration and content uniformity. Despite their usefulness regarding the assessment of these attributes, spectroscopic techniques do not fully capture the evolving mechanisms of granule formation and growth. Dynamic imaging techniques come to complement other monitoring tools by providing a clear insight into nucleation, growth and breakage phenomena, thus giving a complete picture of the granulation continuum. Collectively, the introduction of PATs aims to transform simple process monitoring into a thorough process understanding, shedding light on the intricate relationships between CPPs and CQAs. Digital twins integrate real-time PAT data with mechanistic simulations and machine learning algorithms to create virtual representations of manufacturing processes. These systems enable continuous monitoring, process optimization, predictive maintenance, and advanced control strategies. The integration of digital twins with continuous granulation platforms is expected to play a central role in future pharmaceutical manufacturing environments.

The constant accumulation of complex feedback, generated by the ever-increasing use of PATs, leads to an immediate need for more sophisticated and precise data analysis techniques. Modeling emerges as a critical junction between data generation and effective decision making in the pharmaceutical field. Empirical approaches encompass the principles of multivariate analysis and Design of Experiments, aiming at the uncovering of statistical relationships between process parameters and product quality. Even though it offers a rapid and easily interpretable insight into a granulation process, empirical modeling lacks the ability to capture the dynamically evolving landscape of a given process. This limitation is directly addressed using mechanistic data models that strive to explain the complex granulation phenomena through the study of fundamental principles. By describing interparticle interaction mechanisms, population balances and energy conservation, these models contribute to a more sensitive process understanding, yet their use remains limited in established pharmaceutical practice due to their high computational complexity. To address the limitations and integrate the strengths of both modeling approaches, modern data analysis demands the development of hybrid strategies. When it comes to introducing mechanistic process knowledge to a data-driven framework, hybrid models have shown their superior predictive capabilities across varying operating systems, as well as their adaptability to dynamic and continuous production environments. Thus, improving the current models is necessary to monitor the continuous production process. In order to be able to change the batch-mode of granulation process to the continuous process, it is essential to assess and assure the drug quality and safety and thus to deep understanding of the process mechanisms and all the parameters interfering within and affecting it. After all, to guarantee that the pharmaceutical products resulting from continuous manufacturing process meet regulatory requirements and guidelines in terms of quality, an accurate control system for each unit in the line is necessary, indicating the importance of process understanding through the whole steps.

### 4.2. Future Perspectives, Industrial Challenges, and Regulatory Outlook

The effective translation of research findings into the existing industrial practice is hindered by several limitations. Major issues including the varying process configurations and design spaces coupled with the heterogenous study metrics are factors that significantly affect the generalization capabilities of the available research. Furthermore, while a major part of modern research is focused on transitioning from batch to continuous manufacturing with the subsequent implementation of PATs, many studies remain confined only within the laboratory scale. This gap in scale-up research, especially within modeling frameworks, further supports the idea that modern systems lack the essential computational capabilities to analyze the volumes of data generated by industrial processes. Finally, regulatory standardization of modern technological advancements remains a serious uncertainty. In pharmaceutical development novel techniques require not only to be innovative but also to demonstrate safety and efficiency when it comes to monitoring quality attributes that directly affect patients’ health. Collectively, all limitations mentioned above highlight the need for greater cross validation throughout the available academic research, combined with proper regulatory harmonization across all newly developed techniques.

Most recently, ICH Q13, which specifically addresses the scientific and regulatory considerations for continuous manufacturing of drug substances and drug products. ICH Q13 provides harmonized guidance on the design, implementation, operation, control strategy, and lifecycle management of integrated continuous manufacturing systems, facilitating the transition from conventional batch processing to continuous production while promoting global regulatory convergence [68]. Regulatory authorities increasingly recognize that appropriately validated process models, combined with in-line and on-line analytical measurements, can provide sufficient assurance of product quality, thereby reducing reliance on conventional end-product testing and improving manufacturing efficiency. This approach aligns with lifecycle process verification and continuous process improvement principles promoted throughout the ICH Quality guidelines series.

Recent regulatory initiatives have also acknowledged the growing role of advanced modeling, artificial intelligence (AI), digital twins, and model-based process control in pharmaceutical manufacturing. The FDA’s 2004 Guidance for Industry on Process Analytical Technology (PAT) established a framework for process understanding, monitoring, and control based on timely measurements of critical process and quality attributes, encouraging the pharma industry to build quality into their manufacturing processes rather than relying solely on end-product testing [69].

More recent FDA initiatives have expanded the regulatory focus toward innovative manufacturing technologies. The CDER Framework for Regulatory Advanced Manufacturing Evaluation (FRAME) initiative was established to facilitate the evaluation and support the adoption of the emerging advanced manufacturing technologies, including four core technology priorities: (i) End-to-End Continuous Manufacturing (E2E CM) with a fully integrated process into which raw materials are continuously fed and transformed and finished drug products are continuously removed; (ii) distributed manufacturing (DM) with a decentralized manufacturing strategy with manufacturing units that can be deployed to multiple locations; (iii) distributed manufacturing units at non-traditional host sites, for example, at healthcare facilities; and (iv) artificial intelligence (AI) including software and hardware systems that can perceive the environment, interpret data, and decide actions [70]. In addition, the FDA Advanced Manufacturing Technologies Designation Program provides a regulatory pathway for eligible advanced manufacturing technologies, and accelerates the development and adoption of innovative drug manufacturing methods to improve supply chain resilience, enhance product quality, and prevent drug shortages [71]. These initiatives highlight the increasing regulatory emphasis on transparency, model credibility, data integrity, and lifecycle management when advanced digital technologies are incorporated into pharmaceutical manufacturing. Future regulatory frameworks are expected to further support the implementation of these technologies and autonomous process control, provided that these technologies are appropriately validated, scientifically justified, and integrated within a robust pharmaceutical quality system.

Unfortunately, the relationships among CMAs, CPPs and CQAs are still frequently established through empirical correlations rather than causally grounded mechanistic frameworks. This limits the transferability of process knowledge across scales, equipment platforms, and formulations, particularly in the context of continuous manufacturing. A characteristic example is the incomplete mechanistic description of granulation phenomena, liquid bridge formation, capillary-driven coalescence, the presence of irregular particle morphologies and binder-mediated plastic deformation, lacking also by measurable transition ranges. As a result, many reported CPP effects lack information, limiting predictive process understanding [72,73]. Moreover, the ability of modern PAT tools to monitor process trajectories has not yet been fully integrated into QbD methodologies, omitting the fact that granulation exhibits dynamic process evolution, with critical dynamic phenomena such as nucleation bursts, agglomerate restructuring, and attrition–growth competition, all of which can strongly influence final granule. Although PATs improve observability, they do not necessarily increase understanding, exhibiting limitations for mechanistic interpretability. Thus, PAT data must increasingly be integrated with physics-based or hybrid modeling frameworks to avoid purely correlative decision-making. PATs should be dynamically involved in active real-time control and not only monitoring [57,74].

Furthermore, as regards the modeling approaches, they unfortunately remain fragmented across scales, being sometimes computationally prohibitive for industrial-scale systems, and without being validated yet. The absence of a fully unified multiscale framework remains a major drawback in predictive granulation science, with the hybrid/combination approaches being a possible future solution [75,76]. The need of stochastic design spaces and risk-based modeling frameworks is increasingly recognized by the literature, particularly for continuous manufacturing systems, due to the faced variability in raw material properties, including batch-to-batch differences in particle size distribution, surface energy, moisture sorption behavior, process noises, etc., leading eventually to integrated process models and holistic process understanding across unit operations [57,74].

Scale-up remains a persistent translational challenge, limiting the process transferability despite advances in QbD, mainly due to the potential changes in shear environment, mixing efficiency, and residence time distribution during scale-up, particularly being observed in high-shear and twin-screw granulation, thus leading to non-linear shifts in granule growth behavior. From the materials point of view, the CMAs should not be treated as static variables, while they actually possess a dynamic wettability behavior, with their properties like polymorphism, surface roughness, amorphous content being evolved during granulation due to mechanical stress, solvent exposure, and thermal effects [72,74,77].

Future advancements in granulation processing are strongly governed by continuous manufacturing paradigms and data-driven frameworks. Looking ahead, tighter integration of granulation techniques with modern PATs and efficient modeling tools is expected to be the focus of future academic research. Collectively, digital twins, Explainable AI, hybrid modeling, and RTRT represent key enabling technologies for the next generation of intelligent pharmaceutical manufacturing. Their integration within continuous granulation processes is expected to significantly improve process robustness, manufacturing flexibility, and product quality while supporting the industry’s transition towards fully digitalized and autonomous production systems, automatically compensating for process variability, and ensuring consistent product quality with minimal operator intervention. However, advancements in granulation cannot solely rely on technological innovations without the proper establishment of regulatory frameworks to support robust product quality and sustainable pharmaceutical manufacturing. The future of pharmaceutical granulation lies in the convergence of multiscale mechanistic modeling, advanced PAT integration, and autonomous control systems within a unified digital manufacturing ecosystem. Such strategy remains a challenge not only to get implemented by the pharmaceutical manufacturing sector, but also to normally incorporated and eventually be adapted by respective regulatory frameworks, along with the necessary model validation, cybersecurity, and data management.

Although continuous pharmaceutical manufacturing offers significant advantages, including improved process control, reduced production times, lower material waste, and enhanced product quality, its widespread industrial implementation remains limited. A major challenge lies in integrating upstream and downstream unit operations, such as granulation, drying, milling, and tableting, into a stable, continuous process while maintaining consistent material flow and product quality. Variability in raw material properties, including particle size, moisture content, and flowability, can further affect process performance and requires robust formulations and adaptive control strategies. The successful implementation of PAT also depends on reliable sensor performance, robust chemometric models, and effective management of large process datasets. Although advanced modeling approaches, digital twins, and artificial intelligence have improved process understanding and predictive control, their industrial adoption remains constrained by model validation requirements, computational complexity, and integration with existing manufacturing infrastructure. Economic considerations, including the high capital investment required for new equipment, automation systems, and digital technologies, present additional barriers, particularly for facilities designed for conventional batch processing. From a regulatory perspective, recent guidance such as ICH Q13 provides scientific and regulatory principles for the development, implementation, operation, and lifecycle management of continuous manufacturing for drug substances and drug products; however, manufacturers must still demonstrate robust control strategies, data integrity, and model reliability. Overcoming these technical, economic, organizational, and regulatory challenges will be essential for the broader implementation of continuous pharmaceutical manufacturing and the transition towards Pharma 4.0.

Ultimately, the convergence of Pharma 4.0, artificial intelligence, and continuous manufacturing is poised to redefine pharmaceutical granulation, leading to more efficient, sustainable, flexible, and patient-centered manufacturing processes. Thus, the continuous collaboration between academia, industry, equipment manufacturers, and regulatory agencies is necessary to accelerate the implementation of these technologies.

## 5. Conclusions

Technological advancements in pharmaceutical analysis have shifted the focus from merely optimizing equipment configurations to developing a deeper understanding of the fundamental mechanisms of granulation processes. International regulatory agencies align with this progress by emphasizing Critical quality attributes and critical process parameters according to the paradigms of Quality by Design.

Pharmaceutical manufacturing is increasingly oriented towards continuous processing, exploring alternatives in all granulation categories. These innovations seek not only to improve efficiency but also to support seamless integration with Process Analytical Technology tools. To interpret the vast volumes of data generated by PATs, advanced modeling approaches have become indispensable. Mechanistic models provide critical insights into particle dynamics, agglomeration behavior, and fluid–solid interactions. When combined in hybrid configurations, these models offer even stronger predictive capabilities, aiding process scale-up, optimization, and control. Artificial intelligence (AI) and machine learning (ML) represent the next frontier in granulation science. Early studies demonstrate their potential to enhance both mechanistic understanding and real-time decision-making. As these approaches mature, their success will depend heavily on rigorous experimental design, representative datasets, and careful integration with existing mechanistic models.

Overall, the future of granulation lies in the combination of advanced PAT tools, mechanistic simulations, and AI-driven analytics capable of transforming both batch and continuous manufacturing. These developments promise more consistent product quality, reduced operational costs, and faster development cycles, ultimately enabling a more flexible, efficient, and scientifically grounded pharmaceutical production paradigm.

## Figures and Tables

**Table 4:** Comparative table of the modeling approaches referred to in [25,26,27,28,29,30,31,32,33,34,35,36,37,38,39,40,41,42,43,44,45,46,47,48,49,50,51,52,53,54,55,56].

Modeling Approach	Principle	Advantages	Limitations	Typical Applications
**Empirical (DoE, PCA, PLS)**	Statistical relationships	Simple, rapid, industrially accepted	Limited extrapolation	Process optimization, PAT, QbD
**Mechanistic (PBM)**	Granule population dynamics	Predicts size evolution	Parameter estimation	Granule growth modeling
**Mechanistic (DEM)**	Particle-scale mechanics	Particle interactions, mixing	Computationally intensive	High-shear and twin-screw granulation
**Mechanistic (CFD)**	Fluid and heat transport	Binder distribution, drying	High computational cost	Equipment design, scale-up
**Hybrid models**	Mechanistic + data-driven	High accuracy, interpretable	More complex implementation	Digital twins, process optimization
**Machine learning**	Data-driven AI algorithms	Nonlinear prediction, adaptive	Requires large datasets, less interpretable	Fault detection, predictive control, RTRT

## Data Availability

No new data were created or analyzed in this study. Data sharing is not applicable to this article.

## Abbreviations

The following abbreviations are used in this manuscript:

QbD	Quality by Design
PATs	Process Analytical Technologies
CPPs	Critical Process Parameters
CQAs	Critical Quality Attributes
APIs	Active Pharmaceutical Ingredients
TSWG	Twin-Screw Wet Granulation
NIR	Near-Infrared Spectroscopy
RAG	Resonant Acoustic Wet Granulation
PLS	Chemometric Partial Least Squares
PCA	Principal Component Analysis
BD & TD	Bulk and Tapped Density
PSD	Particle Size Distribution
FBG	Fluid-Bed Granulation
HME	Hot-Melt Extrusion
MDT	Mean Dissolution Time
SF	Solid Fraction
TS	Tensile Strength
MLR	Multiple linear regression
TSMG	Twin-Screw Melt Granulation
AI	Artificial Intelligence
HSWG	High-Shear Wet Granulation
THz-TDS	Terahertz Time-Domain Spectroscopy
SFV	Spatial Filter Velocimetry
DIA	Dynamic Image Analysis
HSI	Hyperspectral Imaging
PBM	Population Balance Model
CFD	Computational Fluid Dynamics
DEM	Discrete Element Method
MTR	Mixer Torque Rheometer
MCC	Microcrystalline Cellulose
RMSEC	Root Mean Square Error of Calibration
RMSECv	Root Mean Square Error of Cross-Validation
RMSEP	Root Mean Square Error of Prediction
